# Supplementary material for: Complexome profiling of the Chlamydomonas psb28 mutant reveals TEF5 as an early PSII assembly factor
Source: Plant Cell. 2025 Mar 17;37(6):koaf055. doi: 10.1093/plcell/koaf055 (PMC12164590; doi:10.1093/plcell/koaf055)
Supplement: koaf055_Supplementary_Data [file koaf055_supplementary_data.zip › psb28_interactive.zip!/plots/intensity/qualityPlots/peptides/PSBP3_Cre12.g509050.t1.1.html]

### Cre12.g509050.t1.1

Trivial name: PSBP3  
SeqCoverage [median]: 54.60  
Mass: 23.57 kDa  
  
 

| Sequence | MissedCleavage | Mass [Da] | wtIntensity | muIntensity | Score |
| --- | --- | --- | --- | --- | --- |
| DITEFGDLATVAETLAK | 0 | 1792.91 | 621.75 | 0.00 | 171.29 |
| DVLTAPGTEVK | 0 | 1128.60 | 6960.20 | 91381.30 | 162.49 |
| EKLETTVK | 1 | 946.53 | 0.00 | 1073.31 | 85.68 |
| FVYPFGWQEVAVK | 0 | 1568.80 | 0.00 | 7576.20 | 71.89 |
| GADVVFK | 0 | 734.40 | 0.00 | 2789.73 | 83.61 |
| GFNPVEDAQDNYR | 0 | 1523.66 | 10186.58 | 85797.62 | 288.11 |
| GHNYYQIEFTASNSR | 0 | 1785.81 | 542.99 | 54092.79 | 139.64 |
| HQLAVVAANNGTFYTLTTGSNER | 0 | 2463.21 | 0.00 | 14497.95 | 142.93 |
| IIATEQR | 0 | 829.47 | 8465.59 | 141089.47 | 173.38 |
| IIATEQREAK | 1 | 1157.64 | 0.00 | 1067.84 | 96.34 |
| MKEKLETTVK | 2 | 1205.67 | 1542.03 | 20564.51 | 104.30 |
|  |  |  | 28319.2 | 419930.7 |  |
